# SNAP judgments into the digital age: Reporting on food stamps varies significantly with time, publication type, and political leaning

**DOI:** 10.1371/journal.pone.0229180

**Published:** 2020-02-21

**Authors:** Benjamin W. Chrisinger, Eliza W. Kinsey, Ellie Pavlick, Chris Callison-Burch

**Affiliations:** 1 Stanford Prevention Research Center, Department of Medicine, Stanford University, Palo Alto, California, United States of America; 2 Department of Epidemiology, Columbia University Mailman School of Public Health, New York, New York, United States of America; 3 Department of Computer Science, Brown University, Providence, Rhode Island, United States of America; 4 Department of Computer and Information Science, University of Pennsylvania, Philadelphia, Pennsylvania, United States of America; University of North Texas Health Science Center, UNITED STATES

## Abstract

The Supplemental Nutrition Assistance Program (SNAP) is the second-largest and most contentious public assistance program administered by the United States government. The media forums where SNAP discourse occurs have changed with the advent of social and web-based media. We used machine learning techniques to characterize media coverage of SNAP over time (1990–2017), between outlets with national readership and those with narrower scopes, and, for a subset of web-based media, by the outlet’s political leaning. We applied structural topic models, a machine learning methodology that categorizes and summarizes large bodies of text that have document-level covariates or metadata, to a corpus of print media retrieved via LexisNexis (n = 76,634). For comparison, we complied a separate corpus via web-scrape algorithm of the Google News API (2012–2017), and assigned political alignment metadata to a subset documents according to a recent study of partisanship on social media. A similar procedure was used on a subset of the print media documents that could be matched to the same alignment index. Using linear regression models, we found some, but not all, topics to vary significantly with time, between large and small media outlets, and by political leaning. Our findings offer insights into the polarized and partisan nature of a major social welfare program in the United States, and the possible effects of new media environments on the state of this discourse.

## Introduction

The Supplemental Nutrition Assistance Program (SNAP, formerly known as the Food Stamp Program) is the United States federal government’s primary form of food assistance to lower-income Americans, and is the second-largest welfare program, with a budget of more than $68 billion in 2017 [1]. The program, which serves over 46 million individuals, includes means-testing and work requirements, both of which have been the topic of Congressional debate and reform efforts since the program’s formalization under the federal Food Stamp Act of 1964. Following a large program expansion under the 2008 American Recovery and Reinvestment Act (ARRA), the size, scope, and nature of SNAP has been increasingly scrutinized by elected officials. Concerns over program fraud, unhealthy eating, and luxury purchasing have also punctuated recent ethical and administrative debates about SNAP [2]. The federal budget proposed by the Trump Administration in February 2018 would codify many of the earlier policy proposals of conservative lawmakers, such as increased work requirements, devolution of funding to state agencies based on block-grant formulas, and significant budget cuts.

While the tenor of policymaking debates over SNAP has shifted since the program’s formation in 1964, many key points of dissention are recurrent themes. For instance, frequent debates include whether or not the program disincentivizes work, encourages welfare dependency, or subsidizes unhealthy eating [3,4]. The tenor of these political debates takes on new dimensions in the modern era, where one in ten households receives SNAP benefits and millions more are eligible, but not enrolled in the program [1]. Government reports indicate that the program now serves a vital role in preventing severe poverty [3,5], and progressive think-tanks and politicians have pointed to the program’s prevalence among hourly wage-earners, suggesting that private sector employers effectively “subsidize” low employee wages with broad-based categorical welfare programs such as SNAP [6,7].

### Influence of media on policy discourse

Media studies have demonstrated how, through the selection and framing of topics, the news media acts as an agenda-setter—impacting the salience of issues and influencing public opinion and policymakers alike [8–10]. Several studies have specifically examined how recipients of social benefits are portrayed in the media (as deserving or undeserving), and hypothesized how this may also influence public opinion and policymakers [11,12]. A content analysis of editorial pages about perceptions of immigrants on welfare, found that prior to a major welfare reform in 1996, older immigrants were often portrayed as undeserving of federal assistance [11]. After the reform, which restricted immigrants’ access to certain welfare benefits, the number of editorial pieces advocating for the worthiness of older immigrants increased dramatically. The framing of social welfare recipients or other populations targeted by social policies in the media can have significant implications for the decisions of policymakers [13,14].

Typically, content analyses of print journalism have included a sample of articles published in major newspapers; however, with the advent of online journalism and social media, an increasing number of individuals access news via digital platforms. Digital news sources now play a major role in the media sector; a 2016 Pew Research Center survey about news use found that 38% of Americans reported getting news from digital news platforms, which was second only to those who got their news from TV sources [15]. As the media landscape has rapidly shifted, researchers have turned to analyses of popular media-sharing platforms to better understand how news content is disseminated [16]. Given these shifts in news media platforms, a much broader approach—requiring new tools for qualitative content analysis—is needed to understand the full scope of coverage for particular social policies and programs.

This study used a machine learning technique, structural topic modeling, to assess when, where, and how SNAP has been covered by news media. In particular, we were interested in understanding how SNAP was portrayed by different types of news media outlets and considering how media discourse on SNAP could affect public opinion and policymaking related to this large social welfare program.

## Materials and methods

**Fig 1:**
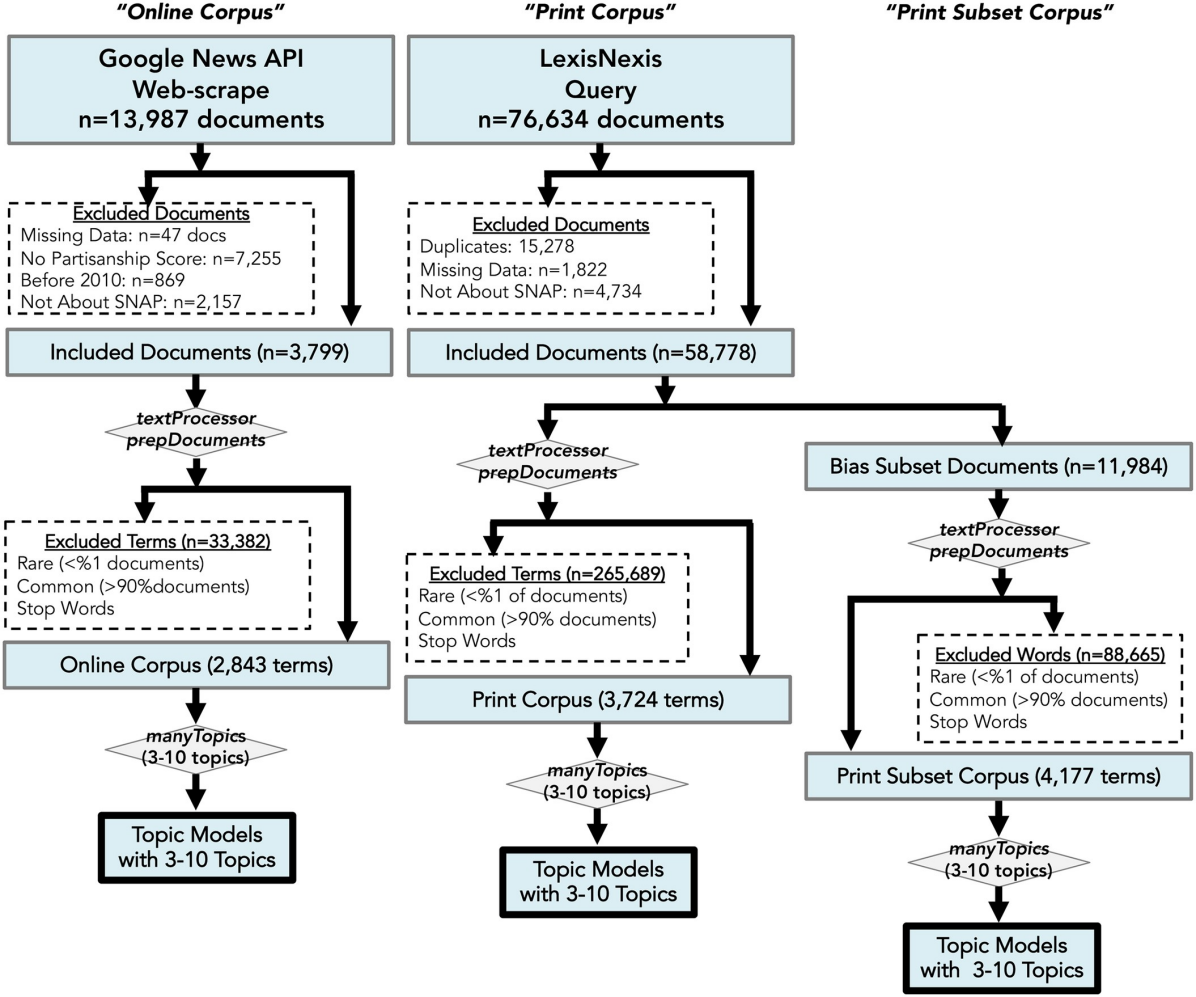
Study flow diagram including data collection, processing, and analysis.

This study was completed in three general steps: 1) assembling corpora of online and print media about SNAP, 2) refining and preparing these databases for statistical analyses using structural topic models to account for possible temporal variation in topics [17], and 3) identifying topics and estimating their prevalence according to different document metadata, such as the date of publication. Fig 1 provides a graphical representation of these steps.

### Corpus assembly

A corpus of print news media (subsequently called the “Print Corpus”) was assembled from a series of queries to the LexisNexis Academic Newspaper Stories/Combined Papers archive, which includes 2,702 major news publications in the United States and elsewhere. Given the size of the archive, we applied simple primary search terms: “food stamp,” “SNAP,” or “Supplemental Nutrition Assistance Program,” and we also included items classified by LexisNexis under the index term “Food Stamps.” We limited the search to articles published between January 1, 1990 and December 31, 2017.

To create an online media corpus (called the “Online Corpus”), we used a Google News web-scraping algorithm based on several broad search keywords to include all mentions of SNAP in online media from 1990 to the present. Primary inclusion terms were: “food stamps,” “SNAP,” and “Supplemental Nutrition Assistance Program,” with additional search terms to identify media that uses state-specific program names (e.g., “CalFresh” in California). A full list of search terms is available in the Supplemental Materials (S1 File). Additional refining of both media corpora was completed in R with pattern-matching functions (available in the *tidyr* package [18]) to remove documents that contained words that may be related to food assistance (e.g., agriculture, hunger, famine) though ultimately were not about SNAP (e.g., “Farmers experience a cold *snap*” or “*Stamp* out hunger”). To our knowledge, the research procedures described above complied with the terms of service for websites from which data were collected.

### Data reduction

The initial search queries within the LexisNexis database generated 79,481 documents. Of these, 58,778 documents were included in the Print Corpus. For the Online Corpus, the initial web-scrape collected 13,987 documents, and ultimately 3,850 documents were included in the final corpus. Fig 1 illustrates the methods used to compile, reduce, and analyze these datasets.

### Corpus pre-processing

With functions available in the *stm* R package [17], several preprocessing steps were taken to prepare the corpora for modeling, such as removing common English “stopwords” (e.g., “the”), punctuation, numbers, and capitalization, as well as word stemming, which retains word stems and discards non-stem parts such as “ing,” “ed,” or “s” (e.g., stemming yields “shop” for “shopping,” “shopped,” and “shops”). We also removed “rare” terms, occurring in less than 1% of all documents in a corpus, as well as common term found in more than 90% of corpus documents. Finally, documents with missing data were removed.

### Metadata extraction and assignment

To allow qualitative investigation of change over time, as well as comparison between media sources and outlets, various document metadata were attached to all documents. For both the Print and Online Corpora, the date of publication was included as metadata, as well as the source’s status as a “Major Paper” or “Other Paper” based on the URLs of the 50 most widely-circulated print newspaper URLs, as reported in a recent Pew Research Center report [15]. This allowed us to introduce a general proxy for national versus local/regional influence or reputation, especially within the Online Corpus, given previous study findings regarding false or misleading “news” by some web content providers [19]. To consider the partisan nature of SNAP coverage, we assigned a “partisanship score” to the Online Corpus ranging from -1.0 (very liberal) to 1.0 (very conservative) [20], based on prior work by Bakshy and colleagues’ [16] assessment of the 500 most prevalent “hard content” URLs shared on Facebook. To apply this same partisanship measure to the Print Corpus, we assigned scores to all “Major Paper” sources (n = 21) that were able to be matched to the Bakshy et al. URLs. This subset of the Print Corpus (“Print Subset Corpus”) included 11,984 documents.

### Statistical analyses

Machine learning methods are generally characterized in one of two ways: supervised or unsupervised analysis. In supervised machine learning analyses, pre-classified documents or data are used to “train” a computer algorithm that can then classify new, un-classified data. These analyses can be useful when distinct categories or typologies are known, *a priori*. Alternatively, unsupervised learning analyzes patterns within a text corpus to identify a given number of themes or categories. This approach is ideal for handling large datasets that do not inherently have well-defined categories, and/or would be prohibitively costly in terms of time or other resources to pre-classify. Here, we apply a particular unsupervised machine learning approach, the “structural topic model,” which has been previously used in analyses of large text databases related to political discourse and attitudes [21]. A specialized R statistical package, *stm*, allows for automated selection of optimal model parameters within a set number of topics, and enables flexible estimation of metadata effects (e.g., changes over time or between liberal and conservative media sources) on topic prevalence [17].

One of the primary outputs of structural topic models are estimates of topic proportions for each document (*θ*), whereby each document is associated with *n* estimates, where *n* is the number of topics in the model. These estimates can be used to assign topics to documents or assess the general distribution of topics across a corpus. The *manyTopics* function (part of the *stm* package) identifies an optimal model for a given number of topics, though inspection of average topic *semantic coherence* (i.e., the relatedness of words within a topic to one another) and *exclusivity* (i.e., the extent to which a topic contains words that do not appear in other topics) for each model was also used to assess the optimal number of topics across all models in a corpus.

For both the Print and Online Corpora, a series of structural topic models were generated for three to ten possible topics with the date of publication and a binary variable for major newspapers (based on the aforementioned Pew study [15]) included as metadata. For the Online Corpus and Print Subset Corpus, a continuous variable for average political alignment was also included as metadata. Descriptive statistics were generated for all expected topic proportions (*θ*), and documents were assigned to topics by their maximum *θ* value. The *stm* package helps summarize each topic by representative words according to several measures: probability, FREX (i.e., its frequency and exclusivity), “lift” (i.e., its weighted frequency in one topic relative to its frequency in other topics), and “score” (i.e., its log-frequency in one topic relative to its log-frequency in other topics) [22]. Documents with the highest *θ* values for a given topic were also inspected to better understand the topics indicated by the representative words. The estimated prevalence of topics and effects of the various metadata were measured with linear regressions fit by the *estimateEffects* function of *stm* [17]. All statistical estimations used 95% confidence intervals and are summarized as Supplemental Material (S2 Appendix).

Eight-topic models were selected for the Print, Print Subset and Online Corpora, based on their average topic semantic coherence and exclusivity, compared to models with higher or lower numbers of topics. These values for all numbers of topics are provided as Supplemental Material (S1 Table).

## Results

### Topics identified

Table 1 provides a full summary of key words from all topic models, as well as the distribution of topics across different document characteristics. Topics in the Print Corpus were related to administrative and community notices (Topic 1, FREX: shall, applic, section, appropri, lifelin, elig, service; and Topic 3, FREX: saturday, chico, church, club, noon, librari, ave), crime reports (Topic 5, FREX: polic, sentenc, court, judg, guilti, prison, arrest), poverty rates (Topic 2, FREX: welfar, poverti, worker, percent, wage, incom, unemploy) and poverty experiences or human interest stories (Topic 8, FREX: got, mother, son, daughter, didnt, apart, feel), federal budget (Topic 4, FREX: budget, billion, tax, cut, senat, spend, bill) and national politics (Topic 7, FREX: immigr, polit, romney, voter, elect, campaign, obama), and agriculture and produce (Topic 6, FREX: farmer, market, veget, land, design, critic, fruit). In the Print Subset Corpus, several similar topics emerged, and were also related to national politics and campaigns (Topic 1, FREX: trump, polit, dole, candid, elect, romney, voter), poverty rates (Topic 4, FREX: wage, rate, economi, percent, poverti, unemploy, minimum), crime reports (Topic 5, FREX: stolen, polic, arrest, prison, sentenc, vehicl, male), poverty experiences or human interest stories (Topic 6, FREX: daughter, neediest, donat, brooklyn, chariti, rent, son), and federal budget (Topic 8, FREX: billion, budget, bill, deficit, farm, medicar, cut). Unique topics in the Print Subset were related to benefit administration and legal requirements (Topic 2, FREX: mayor, citi, council, lawyer, court, file, office), welfare reform (Topic 3, FREX: welfar, recipi, benefit, child, elig, assist, program), and experiences “on food stamps” (Topic 7, FREX: tell, know, didnt, stori, your, got, ive). Similar topics in the Online Corpus, topics included the federal budget (Topic 5, FREX: democrat, republican, senat, vote, bill, trump, ryan), poverty rates (Topic 6, FREX: poverti, wage, tax, earn, rate, econom, minimum), and experiences “on food stamps” (Topic 8, FREX: thing, think, stori, know, realli, dont, tell); unique topics were related to fraud, especially a high-profile case brought by federal prosecutors (Topic 1, FREX: fraud, investig, jeff, alleg, attorney, prison, arrest), anti-hunger programs (Topic 2, FREX: student, hunger, school, meal, pantri, insecur, lunch), SNAP retailers and shopping (Topic 3, FREX: fruit, veget, market, obes, fresh, healthi, soda), work requirements (Topic 4, FREX: main, requir, waiver, able-bodi, test, train, governor), and welfare eligibility and immigrants (Topic 7, FREX: medicaid, elig, immigr, care, servic, grant, military).

**Table 1:** Summary of 8-topic models for Print, Print Subset and Online media Corpora.

#	identifying terms	Count^1^	Year^2^	Major paper^3^	Alignment ^2^^,^^4^
*Print Corpus*					
*1*	FREX: shall, applic, section, appropri, lifelin, elig, servic	3996 (7%)	2012.3 (5.5)	163 (4%)	n/a
	Highest Prob: servic, provid, program, state, requir, inform, assist				
	Lift: lifelin, pursuant, datetim, paragraph, shall, dhs, vacanc				
	Score: shall, section, lifelin, servic, applic, pursuant, appropri				
*2*	FREX: welfar, poverti, worker, percent, wage, incom, unemploy	12413 (21%)	2007.4 (7.5)	2852 (23%)	n/a
	Highest Prob: said, program, people, famili, percent, state, work				
	Lift: low-wag, afdc, minimum-wag, able-bodi, hunger, poverti, census				
	Score: said, poverti, percent, welfar, famili, wage, program				
*3*	FREX: saturday, chico, church, club, noon, librari, ave	4663 (8%)	2009.7 (5.9)	238 (5%)	n/a
	Highest Prob: will, center, free, communiti, call, school, church				
	Lift: bingo, crafter, paradis, presbyterian, amnoon, chico, methodist				
	Score: chico, ave, vallejo, church, noon-, amnoon, noon				
*4*	FREX: budget, billion, tax, cut, senat, spend, bill	7779 (13%)	2007.0 (7.8)	2302 (30%)	n/a
	Highest Prob: tax, budget, bill, year, cut, hous, said				
	Lift: billion, subcommitte, veto, boehner, lawmak, stimulus, budget				
	Score: billion, republican, budget, tax, senat, democrat, said				
*5*	FREX: polic, sentenc, court, judg, guilti, prison, arrest	4164 (7%)	2008.8 (7.8)	720 (17%)	n/a
	Highest Prob: court, state, polic, case, charg, report, offic				
	Lift: defraud, plead, guilti, probat, prosecutor, indict, conspiraci				
	Score: court, sentenc, polic, guilti, plead, probat, arrest				
*6*	FREX: farmer, market, veget, land, design, critic, fruit	2437 (4%)	2011.9 (4.9)	201 (8%)	n/a
	Highest Prob: market, area, farmer, unit, critic, design, use				
	Lift: obes, grain, soil, shopper, wheat, dairi, veget				
	Score: farmer, market, farm, veget, critic, agricultur, fruit				
*7*	FREX: immigr, polit, romney, voter, elect, campaign, obama	10714 (18%)	2008.6 (7.3)	2336 (22%)	n/a
	Highest Prob: presid, american, people, will, one, countri, govern				
	Lift: mitt, romney, poll, politician, gingrich, racism, racist				
	Score: obama, republican, romney, trump, democrat, polit, vote				
*8*	FREX: got, mother, son, daughter, didnt, apart, feel	12612 (21%)	2006.5 (7.5)	3172 (25%)	n/a
	Highest Prob: said, get, say, people, year, one, work				
	Lift: shes, grandmoth, smile, neediest, couch, dad, daughter				
	Score: said, say, get, dont, got, just, daughter				
Overall		58778	2008.2 (7.4)	11984 (20%)	n/a
*Print Subset Corpus*					
*1*	FREX: trump, polit, dole, candid, elect, romney, voter	1427 (12%)	2004.9 (8.9)	1427 (100%)	-0.33 (0.20)
	Highest Prob: presid, said, republican, polit, democrat, american, campaign				
	Lift: santorum, elector, mitt, romney, strategist, pollster, incumb				
	Score: republican, democrat, romney, obama, dole, clinton, senat				
*2*	FREX: mayor, citi, council, lawyer, court, file, offici	1523 (13%)	2002.8 (8.6)	1523 (100%)	-0.34 (0.21)
	Highest Prob: said, citi, state, new, offici, servic, offic				
	Lift: plaintiff, lawsuit, improp, fingerprint, bloomberg, rico, puerto				
	Score: citi, immigr, offici, mayor, court, agenc, depart				
*3*	FREX: welfar, recipi, benefit, child, elig, assist, program	1933 (16%)	1999.9 (8.0)	1933 (100%)	-0.33 (0.17)
	Highest Prob: welfar, program, state, children, work, benefit, people				
	Lift: welfare—work, able-bodi, afdc, caseload,—wedlock, unmarri, childless				
	Score: welfar, children, recipi, program, state, benefit, reform				
*4*	FREX: wage, rate, economi, percent, poverti, unemploy, minimum	1511 (13%)	2005.9 (8.8)	1511 (100%)	-0.35 (0.19)
	Highest Prob: percent, year, incom, job, poverti, rate, increas				
	Lift: economist, census, stagnat, inequ, wage, richest, capita				
	Score: percent, poverti, incom, wage, economi, econom, economist				
*5*	FREX: stolen, polic, arrest, prison, sentenc, vehicl, male	556 (5%)	2000.5 (8.8)	556 (100%)	-0.23 (0.16)
	Highest Prob: counti, block, polic, will, two, school, resid				
	Lift: ave, baton, robberi, stolen, honolulu, roug, lane				
	Score: stolen, block, counti, polic, ave, arrest, man				
*6*	FREX: daughter, neediest, donat, brooklyn, chariti, rent, son	1357 (11%)	2004.3 (8.2)	1357 (100%)	-0.42 (0.16)
	Highest Prob: said, famili, new, children, help, york, month				
	Lift: attn, payabl, schermerhorn, two-bedroom, one-bedroom, archdioces, joralemon				
	Score: neediest, brooklyn, mother, children, famili, daughter, chariti				
*7*	FREX: tell, know, didnt, stori, your, got, ive	2025 (17%)	2004.6 (8.7)	2025 (100%)	-0.33 (0.21)
	Highest Prob: one, say, people, get, like, just, can				
	Lift: yeah, gonna, okay, aint, cup, hey, funni				
	Score: know, think, kid, like, dont, man, thing				
*8*	FREX: billion, budget, bill, deficit, farm, medicar, cut	1652 (14%)	2001.8 (8.3)	1652 (100%)	-0.33 (0.17)
	Highest Prob: bill, budget, tax, cut, billion, hous, program				
	Lift: discretionari, across—board, r-kan, r-ga, r-ohio, billion, veto				
	Score: billion, republican, senat, budget, tax, democrat, vote				
Overall		11984	2003.2 (8.7)	11984 (100%)	-0.34 (0.19)
*Online Corpus*					
*1*	FREX: fraud, investig, jeff, alleg, attorney, prison, arrest	563 (15%)	2015.5 (1.2)	137 (24%)	0.05 (0.49)
	Highest Prob: stamp, fraud, said, card, benefit, charg, store				
	Lift: conspir, lds, lyle, marijuana, mormon, polygamist, theft				
	Score: fraud, flds, prosecutor, indict, investig, lyle, card				
*2*	FREX: student, hunger, school, meal, pantri, insecur, lunch	426 (11%)	2015.3 (1.3)	149 (35%)	-0.33 (0.34)
	Highest Prob: said, school, children, hunger, citi, meal, counti				
	Lift: librari, student, campus, elementari, lunch, pantri, thanksgiv				
	Score: school, pantri, student, insecur, hunger, meal, counti				
*3*	FREX: fruit, veget, market, obes, fresh, healthi, soda	417 (11%)	2015.4 (1.4)	83 (20%)	-0.17 (0.50)
	Highest Prob: market, snap, store, program, farmer, purchas, healthi				
	Lift: dietari, fruit, junk, poultri, calori, desert, shopper				
	Score: farmer, store, veget, market, fruit, healthi, obes				
*4*	FREX: main, requir, waiver, able-bodi, test, train, governor	549 (14%)	2015.6 (1.0)	139 (25%)	0.00 (0.58)
	Highest Prob: state, stamp, work, said, requir, people, benefit				
	Lift: abawd, reinstat, able-bodi, job-train, three-month, waiver, lepag				
	Score: waiver, able-bodi, welfar, job, work, requir, unemploy				
*5*	FREX: democrat, republican, senat, vote, bill, trump, ryan	408 (11%)	2014.5 (1.3)	86 (21%)	-0.31 (0.53)
	Highest Prob: bill, cut, republican, stamp, program, hous, farm				
	Lift: mcgovern, boehner, gop, senat, vote, cotton, gingrich				
	Score: republican, farm, bill, vote, democrat, senat, cut				
*6*	FREX: poverti, wage, tax, earn, rate, econom, minimum	502 (13%)	2015.0 (1.3)	81 (16%)	-0.17 (0.60)
	Highest Prob: poverti, percent, american, incom, tax, year, rate				
	Lift: eitc, index, gdp, means-test, median, census, poverti				
	Score: poverti, wage, welfar, rate, percent, incom, minimum				
*7*	FREX: medicaid, elig, immigr, care, servic, grant, militari	355 (9%)	2015.2 (1.3)	70 (20%)	0.01 (0.57)
	Highest Prob: program, benefit, state, assist, famili, snap, feder				
	Lift: refuge, diaper, ssi, immigr, spous, militari, foster				
	Score: medicaid, elig, servic, benefit, refuge, famili, wic				
*8*	FREX: thing, think, stori, know, realli, dont, tell	579 (15%)	2014.8 (1.3)	77 (13%)	-0.24 (0.57)
	Highest Prob: people, get, like, make, just, one, work				
	Lift: mcdonald, mayb, youd, sick, ive, paltrow, ridicul				
	Score: job, walmart, paltrow, hour, work, dont, think				
Overall		3799	2015.2 (1.3)	822 (22%)	-0.14 (0.55)

Source:

^1^Presented as: N (% of corpus total);

^2^Presented as: mean (SD);

^3^Based on a Pew Research Center identification of the top 50 most widely-circulated media sources (“Major Papers”), presented as N (% of row total).

^4^Based on scores assigned to media URLs by Bakshy et al.’s [16] analysis of content shared on Facebook (<0 more liberal; >0 more conservative).

The average prevalence of topics across Print Corpus documents ranged from 4% (Topic 6, FREX: farmer, market, veget, land, design, critic, fruit) to 21% (Topic 8, FREX: got, mother, son, daughter, didnt, apart, feel). The average year of publication was 2008 (SD = 7.4), and 20% of documents were from newspapers with national readership. Among the Print Subset corpus, the average prevalence ranged from 5% (Topic 5, FREX: stolen, polic, arrest, prison, sentenc, vehicl, male) to 17% (Topic 7, FREX: tell, know, didnt, stori, your, got, ive), and the average political alignment score was -0.34 (SD = 0.19). Online media topics varied in prevalence from an average of 9% (Topic 7, FREX: medicaid, elig, immigr, care, servic, grant, militari), to 15% (Topic 8, FREX: thing, think, stori, know, realli, dont, tell). The average year of publication for Online Corpus documents was 2015 (SD = 1.3), 22% of documents were from major news outlets, and the average political alignment score was -0.14 (SD = 0.55).

### Topic variation

**Fig 2:**
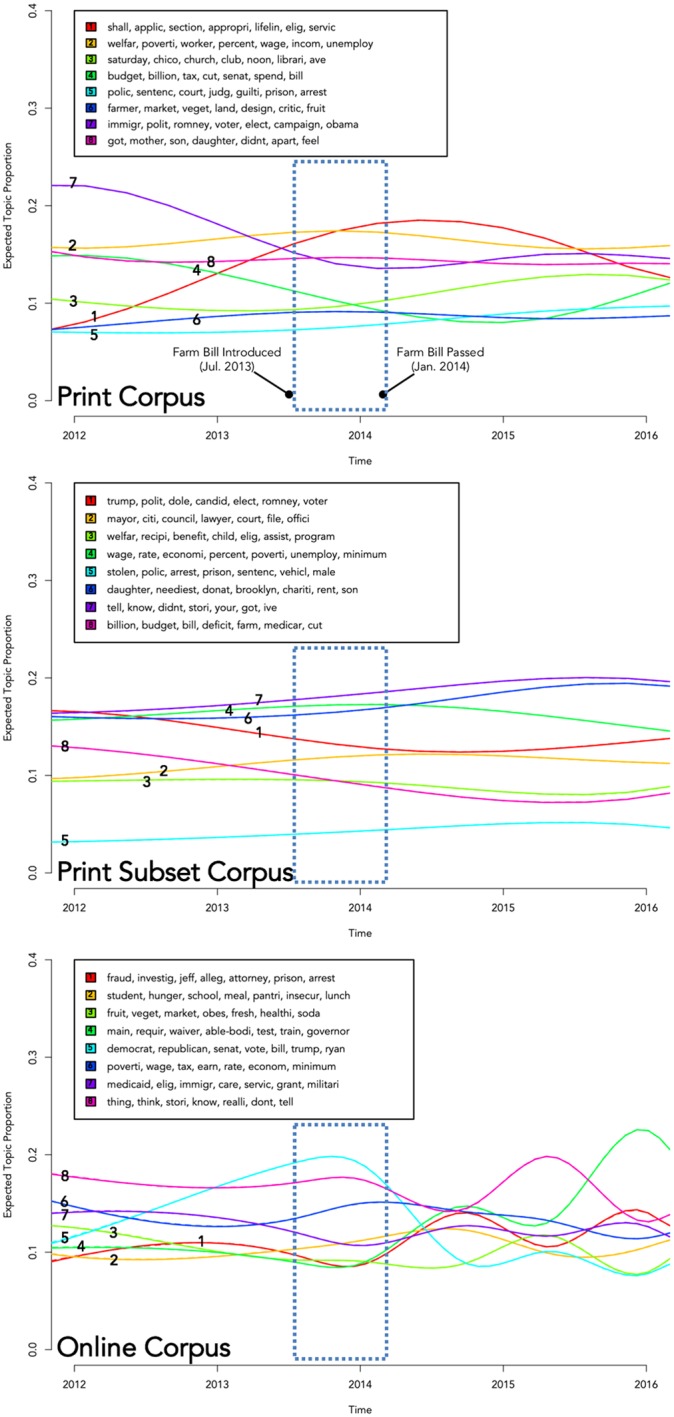
Expected SNAP topic proportions for 8-topic Print and Online media models, 2012–2016. Source: Authors’ analysis of assembled Print and Online media corpora.

To explore the variance of topic proportions over time, both unadjusted and adjusted regression models were fit with parameters from the structural topic models. The expected prevalence of topics was observed to significantly vary between time intervals in all corpora, adjusting for average political alignment (for the Print Subset and Online corpora only) and major news outlet status (for the Print and Online corpora). All topics were found to vary significantly between time periods in the Print Corpus and Print Subset corpora, and five of eight topics had significant time variation in the Online Corpus. Fig 2 provides a visualization of how estimated topic proportions vary within two years of the introduction and passage of the 2014 Farm Bill.

Significant differences between major and non-major media outlets were also observed. Among the Print Corpus, documents from major newspapers had significantly more coverage for half of the topics, and significantly less coverage for the remaining topics. Major media outlets were less predictive of Online Corpus topics and were significantly associated with less coverage for two topics, and more coverage for three topics. Table 2 gives a full summary of these estimates.

**Table 2:** Effect of covariates on estimated topic proportions: Print, Print Subset, and Online Corpora.

Topic	Identifying terms	Major paper^1^^,^^2^	Time^1^^,^^3^	Alignment^1^^,^^4^
*Print Corpus*				
*1*	FREX: shall, applic, section, appropri, lifelin, elig, servic	- ***	- ***	n/a
	Highest Prob: servic, provid, program, state, requir, inform, assist			
	Lift: lifelin, pursuant, datetim, paragraph, shall, dhs, vacanc		+ ***	
	Score: shall, section, lifelin, servic, applic, pursuant, appropri			
*2*	FREX: welfar, poverti, worker, percent, wage, incom, unemploy	+ ***	- ***	n/a
	Highest Prob: said, program, people, famili, percent, state, work			
	Lift: low-wag, afdc, minimum-wag, able-bodi, hunger, poverti, census		+ ***	
	Score: said, poverti, percent, welfar, famili, wage, program			
*3*	FREX: saturday, chico, church, club, noon, librari, ave	- ***	- ***	n/a
	Highest Prob: will, center, free, communiti, call, school, church			
	Lift: bingo, crafter, paradis, presbyterian, amnoon, chico, methodist		+ *	
	Score: chico, ave, vallejo, church, noon-, amnoon, noon			
*4*	FREX: budget, billion, tax, cut, senat, spend, bill	+ ***	+ ***	n/a
	Highest Prob: tax, budget, bill, year, cut, hous, said			
	Lift: billion, subcommitte, veto, boehner, lawmak, stimulus, budget			
	Score: billion, republican, budget, tax, senat, democrat, said			
*5*	FREX: polic, sentenc, court, judg, guilti, prison, arrest	- ***	- ***	n/a
	Highest Prob: court, state, polic, case, charg, report, offic			
	Lift: defraud, plead, guilti, probat, prosecutor, indict, conspiraci			
	Score: court, sentenc, polic, guilti, plead, probat, arrest			
*6*	FREX: farmer, market, veget, land, design, critic, fruit	- ***	- **	n/a
	Highest Prob: market, area, farmer, unit, critic, design, use			
	Lift: obes, grain, soil, shopper, wheat, dairi, veget		+ ***	
	Score: farmer, market, farm, veget, critic, agricultur, fruit			
*7*	FREX: immigr, polit, romney, voter, elect, campaign, obama	+ ***	+ ***	n/a
	Highest Prob: presid, american, people, will, one, countri, govern			
	Lift: mitt, romney, poll, politician, gingrich, racism, racist			
	Score: obama, republican, romney, trump, democrat, polit, vote			
*8*	FREX: got, mother, son, daughter, didnt, apart, feel	+ ***	- ***	n/a
	Highest Prob: said, get, say, people, year, one, work			
	Lift: shes, grandmoth, smile, neediest, couch, dad, daughter			
	Score: said, say, get, dont, got, just, daughter			
*Print Subset Corpus*				
*1*	FREX: trump, polit, dole, candid, elect, romney, voter	n/a	- ***	
	Highest Prob: presid, said, republican, polit, democrat, american, campaign			
	Lift: santorum, elector, mitt, romney, strategist, pollster, incumb		+ ***	
	Score: republican, democrat, romney, obama, dole, clinton, senat			
*2*	FREX: mayor, citi, council, lawyer, court, file, offici	n/a	- ***	+ **
	Highest Prob: said, citi, state, new, offici, servic, offic			
	Lift: plaintiff, lawsuit, improp, fingerprint, bloomberg, rico, puerto		+ ***	
	Score: citi, immigr, offici, mayor, court, agenc, depart			
*3*	FREX: welfar, recipi, benefit, child, elig, assist, program	n/a	- *	
	Highest Prob: welfar, program, state, children, work, benefit, people			
	Lift: welfare—work, able-bodi, afdc, caseload,—wedlock, unmarri, childless		+ ***	
	Score: welfar, children, recipi, program, state, benefit, reform			
*4*	FREX: wage, rate, economi, percent, poverti, unemploy, minimum	n/a	- ***	
	Highest Prob: percent, year, incom, job, poverti, rate, increas		+ *	
	Lift: economist, census, stagnat, inequ, wage, richest, capita			
	Score: percent, poverti, incom, wage, economi, econom, economist			
*5*	FREX: stolen, polic, arrest, prison, sentenc, vehicl, male	n/a	- *	+ ***
	Highest Prob: counti, block, polic, will, two, school, resid			
	Lift: ave, baton, robberi, stolen, honolulu, roug, lane			
	Score: stolen, block, counti, polic, ave, arrest, man			
*6*	FREX: daughter, neediest, donat, brooklyn, chariti, rent, son	n/a	- ***	- ***
	Highest Prob: said, famili, new, children, help, york, month			
	Lift: attn, payabl, schermerhorn, two-bedroom, one-bedroom, archdioces, joralemon			
	Score: neediest, brooklyn, mother, children, famili, daughter, chariti			
*7*	FREX: tell, know, didnt, stori, your, got, ive	n/a	+ **	+ *
	Highest Prob: one, say, people, get, like, just, can			
	Lift: yeah, gonna, okay, aint, cup, hey, funni			
	Score: know, think, kid, like, dont, man, thing			
*8*	FREX: billion, budget, bill, deficit, farm, medicar, cut	n/a	- ***	+ ***
	Highest Prob: bill, budget, tax, cut, billion, hous, program			
	Lift: discretionari, across—board, r-kan, r-ga, r-ohio, billion, veto		+ ***	
	Score: billion, republican, senat, budget, tax, democrat, vote			
*Online Corpus*				
*1*	FREX: fraud, investig, jeff, alleg, attorney, prison, arrest	+ *	+ **	+ ***
	Highest Prob: stamp, fraud, said, card, benefit, charg, store			
	Lift: conspir, lds, lyle, marijuana, mormon, polygamist, theft			
	Score: fraud, flds, prosecutor, indict, investig, lyle, card			
*2*	FREX: student, hunger, school, meal, pantri, insecur, lunch	+ ***	- *	- *
	Highest Prob: said, school, children, hunger, citi, meal, counti			
	Lift: librari, student, campus, elementari, lunch, pantri, thanksgiv			
	Score: school, pantri, student, insecur, hunger, meal, counti			
*3*	FREX: fruit, veget, market, obes, fresh, healthi, soda		+ *	- *
	Highest Prob: market, snap, store, program, farmer, purchas, healthi			
	Lift: dietari, fruit, junk, poultri, calori, desert, shopper			
	Score: farmer, store, veget, market, fruit, healthi, obes			
*4*	FREX: main, requir, waiver, able-bodi, test, train, governor	+ **	+ *	+ ***
	Highest Prob: state, stamp, work, said, requir, people, benefit			
	Lift: abawd, reinstat, able-bodi, job-train, three-month, waiver, lepag			
	Score: waiver, able-bodi, welfar, job, work, requir, unemploy			
*5*	FREX: democrat, republican, senat, vote, bill, trump, ryan		+ *	- ***
	Highest Prob: bill, cut, republican, stamp, program, hous, farm			
	Lift: mcgovern, boehner, gop, senat, vote, cotton, gingrich			
	Score: republican, farm, bill, vote, democrat, senat, cut			
*6*	FREX: poverti, wage, tax, earn, rate, econom, minimum	- *		- ***
	Highest Prob: poverti, percent, american, incom, tax, year, rate			
	Lift: eitc, index, gdp, means-test, median, census, poverti			
	Score: poverti, wage, welfar, rate, percent, incom, minimum			
*7*	FREX: medicaid, elig, immigr, care, servic, grant, militari			+ ***
	Highest Prob: program, benefit, state, assist, famili, snap, feder			
	Lift: refuge, diaper, ssi, immigr, spous, militari, foster			
	Score: medicaid, elig, servic, benefit, refuge, famili, wic			
*8*	FREX: thing, think, stori, know, realli, dont, tell	- ***		- ***
	Highest Prob: people, get, like, make, just, one, work			
	Lift: mcdonald, mayb, youd, sick, ive, paltrow, ridicul			
	Score: job, walmart, paltrow, hour, work, dont, think			

Source:

^1^Presented as: + (positive association),—(negative association),

* (p<0.05),

*** (p<0.001), ns (no significant association);

^2^Based on a Pew Research Center identification of the top 50 most widely-circulated media sources (“Major Papers”), presented as N (% of total);

^3^Categorical time variable of ten time periods, with the first time point set as referent category, thus capturing fluctuations in topic proportions; hence, both positive and negative associations were possible;

^4^Based on scores assigned to media URLs by Bakshy et al.’s [16] analysis of content shared on Facebook (<0 more liberal; >0 more conservative).

**Fig 3:**
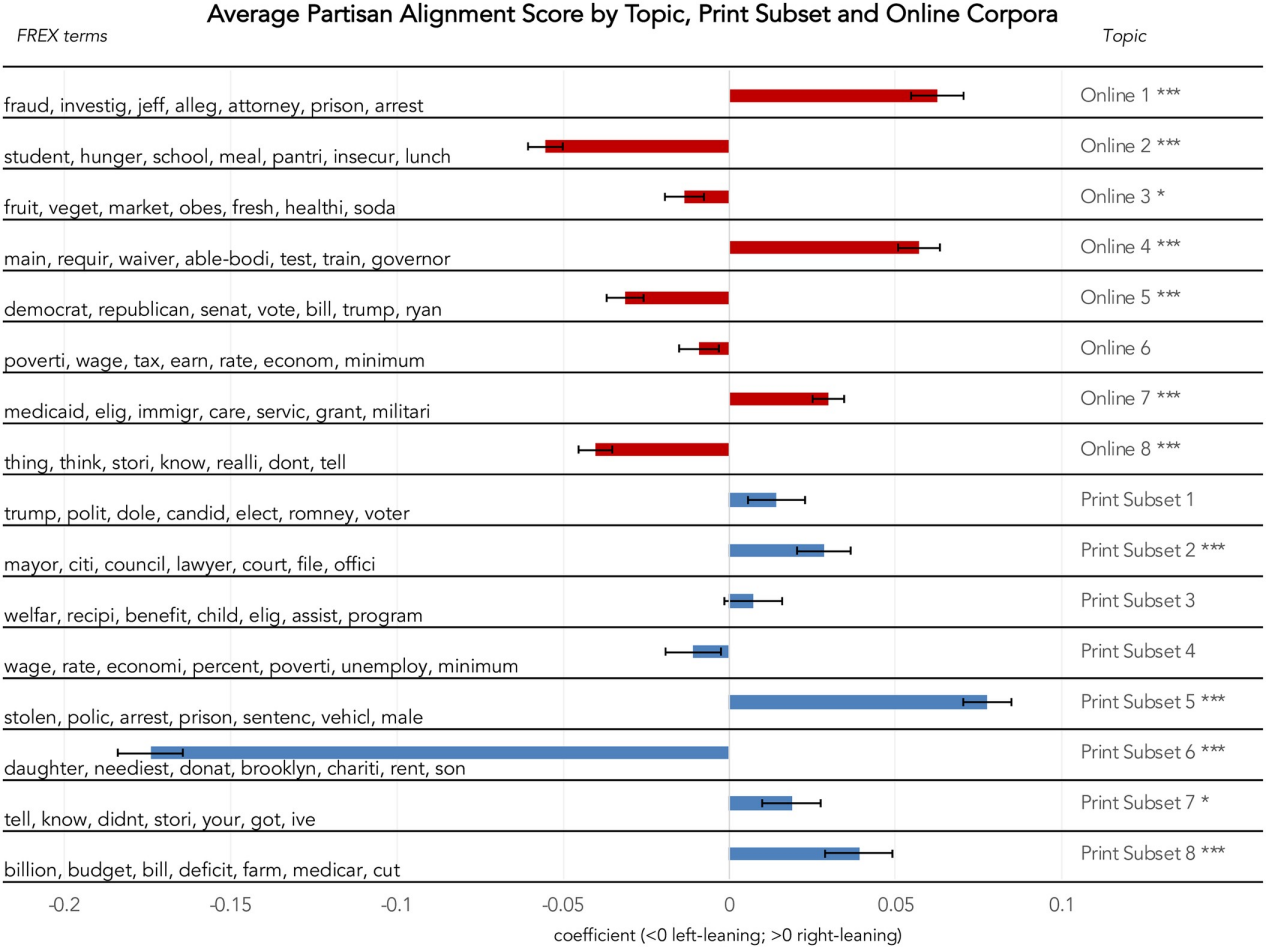
Effect of average partisan alignment on proportion of documents devoted to each topic within the Online and Print Subset Corpora (n = 3,850). Source: Authors’ analysis of assembled media corpora. Political alignment of sources was determined based on scores assigned to media URLs by Bakshy et al.’s [16] analysis of content shared on Facebook. Estimates (bars) and standard errors (lines) presented. * (p<0.05), *** (p<0.001).

For the Online Corpus, the average political alignment of a media source was significantly associated with expected topic coverage: three topics were significantly associated with right-leaning outlets, and five topics with left-leaning outlets. For the Print Subset Corpus, four topics were significantly associated with right-leaning outlets, and one with left-leaning outlets. Fig 3 illustrates the average alignment of Online Corpus topics, along with the highest-probability words within each topic.

### Maximum topic prevalence

On average, maximum topic *θ* values for Print documents were 0.53 (SD = 0.17), 0.48 (SD = 0.15) for Print Subset documents, and 0.49 (SD = 0.14) for Online documents. For “topic-dedicated” documents with a maximum *θ* value of 0.50 or greater (i.e., at least 50% of a document’s text was estimated to pertain to a single topic), a similar distribution is evident between the Print, Print Subset and Online Corpora (average maximum *θ* values 0.68, 0.63, and 0.64, respectively). Among these more focused documents in the Print Corpus, 23% were classified as Print Topic 8 (FREX: got, mother, son, daughter, didnt, apart, feel); in the Print Subset Corpus, 20% were classified as Print Subset Topic 8 (FREX: billion, budget, bill, deficit, farm, medicar, cut), and in the Online Corpus, 27% were classified as Online Topic 1 (FREX: fraud, investig, jeff, alleg, attorney, prison, arrest). Table 3 summarizes the distribution of topics across all documents in greater detail.

**Table 3:** Average maximum prevalence by topic for Print, Print Subset, and Online Corpora.

Topic	FREX terms	Average Maximum Theta		Average Max. Theta > 0.50	
		Count (%)	Mean, SD	Count (%)	Mean, SD
Print Corpus					
1	FREX: shall, applic, section, appropri, lifelin, elig, servic	3996 (7%)	0.63, 0.25	2413 (9%)	0.81, 0.15
2	FREX: welfar, poverti, worker, percent, wage, incom, unemploy	12413 (21%)	0.49, 0.14	5160 (19%)	0.62, 0.09
3	FREX: saturday, chico, church, club, noon, librari, ave	4663 (8%)	0.66, 0.25	2993 (11%)	0.81, 0.17
4	FREX: budget, billion, tax, cut, senat, spend, bill	7779 (13%)	0.50, 0.14	3598 (13%)	0.63, 0.09
5	FREX: polic, sentenc, court, judg, guilti, prison, arrest	4164 (7%)	0.53, 0.18	2182 (8%)	0.67, 0.13
6	FREX: farmer, market, veget, land, design, critic, fruit	2437 (4%)	0.43, 0.12	612 (2%)	0.60, 0.09
7	FREX: immigr, polit, romney, voter, elect, campaign, obama	10714 (18%)	0.48, 0.14	4244 (15%)	0.62, 0.09
8	FREX: got, mother, son, daughter, didnt, apart, feel	12612 (21%)	0.52, 0.15	6411 (23%)	0.65, 0.10
Avg.		7347 (13%)	0.53, 0.17	3452 (13%)	0.68, 0.11
Print Subset Corpus					
1	FREX: trump, polit, dole, candid, elect, romney, voter	1427 (12%)	0.45, 0.13	479 (11%)	0.60, 0.08
2	FREX: mayor, citi, council, lawyer, court, file, offici	1523 (13%)	0.42, 0.11	343 (8%)	0.58, 0.07
3	FREX: welfar, recipi, benefit, child, elig, assist, program	1933 (16%)	0.45, 0.12	637 (14%)	0.59, 0.07
4	FREX: wage, rate, economi, percent, poverti, unemploy, minimum	1511 (13%)	0.47, 0.15	538 (12%)	0.63, 0.10
5	FREX: stolen, polic, arrest, prison, sentenc, vehicl, male	556 (5%)	0.47, 0.18	170 (4%)	0.68, 0.17
6	FREX: daughter, neediest, donat, brooklyn, chariti, rent, son	1357 (11%)	0.57, 0.20	740 (17%)	0.73, 0.11
7	FREX: tell, know, didnt, stori, your, got, ive	2025 (17%)	0.44, 0.14	628 (14%)	0.61, 0.08
8	FREX: billion, budget, bill, deficit, farm, medicar, cut	1652 (14%)	0.53, 0.15	895 (20%)	0.65, 0.09
Avg.		1498 (13%)	0.48, 0.15	554 (13%)	0.63, 0.10
Online Corpus					
1	FREX: fraud, investig, jeff, alleg, attorney, prison, arrest	563 (15%)	0.67, 0.20	436 (27%)	0.76, 0.14
2	FREX: student, hunger, school, meal, pantri, insecur, lunch	426 (11%)	0.46, 0.13	147 (9%)	0.62, 0.08
3	FREX: fruit, veget, market, obes, fresh, healthi, soda	417 (11%)	0.49, 0.14	200 (12%)	0.61, 0.09
4	FREX: main, requir, waiver, able-bodi, test, train, governor	549 (14%)	0.53, 0.16	282 (18%)	0.66, 0.11
5	FREX: democrat, republican, senat, vote, bill, trump, ryan	408 (11%)	0.50, 0.16	162 (10%)	0.67, 0.11
6	FREX: poverti, wage, tax, earn, rate, econom, minimum	502 (13%)	0.45, 0.12	153 (10%)	0.60, 0.08
7	FREX: medicaid, elig, immigr, care, servic, grant, militari	355 (9%)	0.42, 0.11	72 (4%)	0.58, 0.06
8	FREX: thing, think, stori, know, realli, dont, tell	579 (15%)	0.44, 0.12	154 (10%)	0.60, 0.07
Avg.		475 (13%)	0.49, 0.14	201 (13%)	0.64, 0.09

Overall, 46% of Print Media documents predominantly pertained to a single topic (“topic-dedicated”), compared to 37% of Print Subset documents and 42% of Online documents. This indicates that the majority of documents included multiple topics, with a possibility of some documents having almost an equal split of two or more topics. Among certain topics, however, the average document’s maximum theta value was much higher; for example, approximately 77% of the documents classified as Online Topic 1 (FREX: fraud, investing, jeff, alleg, attorney, prison, arrest) were topic-dedicated, but only 20% of Online Topic 7 (FREX: medicaid, elig, immigr, care, servic, grant, militari) documents were similarly topic-dedicated, indicating that the topic more often co-occurred with other topics. These patterns are further detailed in Table 3.

## Discussion

Our findings show how the discourse around SNAP in print and online media significantly varies based on when and where news is reported. Below we highlight two key findings, which may have particularly implications for the broader political discourse and the public perception of SNAP: 1) changes in topic coverage of SNAP over time and in print versus online media, and 2) thematic reporting on SNAP by partisan leaning of news source.

### Changing topic coverage of SNAP in print and online media may influence policymaking

In both the print and online media, we found significant variations over time in topic coverage around SNAP (see Fig 2). These fluctuations were more dramatic for some topics than others. For example, within the Online Corpus, discussion of federal budget (e.g., the Farm Bill) (Online Topic 5), anti-hunger programs (Online Topic 2), SNAP shopping and retailers (Online Topic 3), work requirements (Online Topic 4) and fraud (Online Topic 1) varied significantly, while changes in coverage of the other three topics were less dramatic. Within the Print and Print Subset corpora, all topics varied significantly with time, and many topics were significantly more likely to occur in certain time periods and significantly less likely to occur in others.

As other media content studies have indicated, coverage and framing of a policy issue is often different before versus after a major policy shift [11]. Not surprisingly, within the print corpus we see a significant increase in coverage of the Farm Bill (e.g., Print Topic 4) around both 2008 and 2012—years in which a new Farm Bill was drafted and debated in Congress. Within the online corpus, there is a temporal shift in topic coverage related to policy changes in work requirements (e.g., Online Topic 4) implemented as part of the American Recovery and Reinvestment Act (ARRA). Prior to 2015, state-level work requirement waivers were sunset as part of ARRA, and we notice a marked uptick in Topic 5 coverage in this time period.

Given that SNAP is an income-eligible entitlement program used by one in seven Americans [1,23], it is not surprising that the substantial participant population would generate interest in questions around eligibility (e.g., Online Topic 5). However, while this topic is well represented in online articles (13% of the corpus), deeper investigation suggests that these articles often detail efforts to restrict or curtail eligibility, including proposals to impose drug testing requirements or reinstate work requirements for “able-bodied adults without dependents,” or ABAWDs. Thus, within online media, the topic of eligibility emerges predominantly at times of major policy discussions and often uses negative framing of SNAP recipients, which has implications for policy decisions around issues such as work requirements.

However, shifts in SNAP topic coverage are not just reactions to current events. Given what is known about how media outlets, both print and online, act as agenda-setters for public discourse and for policymakers [8–10,24], the significant variations we found in topic coverage of SNAP may also have implications for direction of SNAP policymaking, as well. The changes in coverage within the Online Corpus of the fraud and abuse topic (a topic in which SNAP recipients are often portrayed in a negative light) are also notable, as they highlight the potential influence of negative media framing on SNAP policy decisions. Alternatively, the episodic nature of certain topics’ media coverage may instead be attributable to Congressional policy schedules, whereby certain topics are perennially brought into the public consciousness via news media as they are more relevant for motivating or achieving political outcomes. Still, it is worth considering media as a mutually reinforcing mechanism by which policymakers’ partisan attitudes and debates are amplified and disseminated to the general public, perhaps generating support or opposition for a particular proposal or idea. This study finds significant variation in the prevalence of topics over time, and that these temporal trends appear stronger for some topics more than others, though it is beyond the scope of our analysis to conclusively determine the direction of the relationship between news media and policy.

### Reporting by partisan leaning of news media source

Based on our analysis, it is evident that the political leaning of media outlets influences the focus of the discussion around SNAP. Within the Online Corpus, all of the topic areas received significantly more coverage in either conservative or liberal media outlets (see Table 2 and Fig 2). Substantively, this looks like more left-leaning media outlets (e.g., Huffington Post or the Washington Post) framing the discussion of SNAP through topics related to poverty rates, federal budget, hunger, SNAP shopping and food access, while more conservative media outlets tend to use the framing of work requirements, program enrollment or eligibility, and fraud.

While public dislike of welfare has been previously documented among Americans [4,12], recent research by political scientist Suzanne Mettler highlights the potency of particular words in shaping public opinion for government-funded social support [25]. According to Mettler’s study, Americans who disliked the term “welfare” tended to be more distrustful of government policy in general, even if they themselves have benefited from some kind of government support (e.g., Medicaid, unemployment insurance). Word choice is powerful and particularly polarizing words such as “welfare” can frame discussions about social programs in a negative light. Indeed, within our study, the word “welfare” was associated with Print Topic 2, Print Subset Topic 3, and Online Topics 4 and 6. Of these topics with political alignment scores, two significant associations were identified: Online Topic 4 (right-leaning) and Online Topic 6 (left-leaning), suggesting that perhaps the intended news audience is also an important dimension to the use of polarizing terminology.

Currently the largest federal food assistance program and one of the largest forms of federally funded financial assistance, SNAP has persistently been the focus of fiscal conservatives. The debate around SNAP has been laden with many of the same contentious issues as with conventional cash welfare including the deservingness of recipients and dependency or abuse of the program by the poor [4,26]. The framing in conservative media outlets of SNAP through the lens of fraud, eligibility and welfare, rather than as a discussion around hunger, poverty and nutrition may also influence the ongoing political debate that surrounds the program.

### Limitations

One major limitation to this study is its chronological focus (1990–2017). While out of necessity given the nature of archived online content and web-scraping technologies, this does limit our ability to monitor changes in topics over broader time horizons. Nevertheless, both the magnitude and reach of online media sources (including traditional media outlets with an online presence and new online-only publications) underscores and mirrors broader shifts in the media landscape, and reinforces our interest in online-accessible documents. Additionally, structural topic models are but one method of assessing large qualitative datasets, and, given their reliance on machine learning, may not always produce externally valid results. However, because we reviewed and qualitatively assessed model outputs, thus refining and enhancing our media corpora based on preliminary findings, we believe that the final topics described have a higher degree of validity.

## Conclusions

We attempted to document the various ways in which the Supplemental Nutrition Assistance Program has been represented in news media during the last several decades. Our findings illustrate how the current era of online media complicates previous content analysis strategies. To adapt to these challenges, we employed a topic modeling procedure that was sensitive to changes over time in order to find internally and externally valid topics. In an eight-topic model, themes included a variety of social, political, and economic issues, with expected topic proportions ranging from approximately 9 to 21 percent, and wide variability for certain topics over time. We also found that SNAP coverage fluctuates or remains steady depending on the topic, and while most news media documents featured at least partial representation of multiple topics, some topics, especially fraud, frequently dominated the documents in which they appeared. Combined with the significant political alignment of most topics, we conclude that certain types of SNAP media coverage are both highly partisan in nature and stand relatively apart from the broader media ecosystem.

## Supporting information

S1 Appendix Summaries for selected 8-topic Print, Print Subset, and Online media models. (DOCX)

S2 Appendix Statistical summary. (DOCX)

S1 Table Semantic coherence and exclusivity summaries for all Print and Online media structural topic models (3 to 10 topics). (XLSX)

S1 File Search terms for online media corpus, including state-specific SNAP programs. (XLSX)

S2 File Underlying dataset for Online Corpus. (CSV)

S3 File Minimal underlying dataset for Print Corpus. (CSV)

S4 File Minimal underlying dataset for Print Subset Corpus. (CSV)

S5 File R Code for data processing, statistical analyses, and figure/table outputs. (R)
