# Supplementary material for: SNAP judgments into the digital age: Reporting on food stamps varies significantly with time, publication type, and political leaning
Source: PLoS One. 2020 Feb 21;15(2):e0229180. doi: 10.1371/journal.pone.0229180 (PMC7034891; doi:10.1371/journal.pone.0229180)
Supplement: S1 Appendix — (DOCX) [file pone.0229180.s001.docx]

> summary(full_snap8)

A topic model with 8 topics, 58778 documents and a 3724 word dictionary.

Topic 1 Top Words:

Highest Prob: servic, provid, program, state, requir, inform, assist

FREX: shall, applic, section, appropri, lifelin, elig, servic

Lift: lifelin, pursuant, datetim, paragraph, shall, dhs, vacanc

Score: shall, section, lifelin, servic, applic, pursuant, appropri

Topic 2 Top Words:

Highest Prob: said, program, peopl, famili, percent, state, work

FREX: welfar, poverti, worker, percent, wage, incom, unemploy

Lift: low-wag, afdc, minimum-wag, able-bodi, hunger, poverti, census

Score: said, poverti, percent, welfar, famili, wage, program

Topic 3 Top Words:

Highest Prob: will, center, free, communiti, call, school, church

FREX: saturday, chico, church, club, noon, librari, ave

Lift: bingo, crafter, paradis, presbyterian, amnoon, chico, methodist

Score: chico, ave, vallejo, church, noon-, amnoon, noon

Topic 4 Top Words:

Highest Prob: tax, budget, bill, year, cut, hous, said

FREX: budget, billion, tax, cut, senat, spend, bill

Lift: billion, subcommitte, veto, boehner, lawmak, stimulus, budget

Score: billion, republican, budget, tax, senat, democrat, said

Topic 5 Top Words:

Highest Prob: court, state, polic, case, charg, report, offic

FREX: polic, sentenc, court, judg, guilti, prison, arrest

Lift: defraud, plead, guilti, probat, prosecutor, indict, conspiraci

Score: court, sentenc, polic, guilti, plead, probat, arrest

Topic 6 Top Words:

Highest Prob: market, area, farmer, unit, critic, design, use

FREX: farmer, market, veget, land, design, critic, fruit

Lift: obes, grain, soil, shopper, wheat, dairi, veget

Score: farmer, market, farm, veget, critic, agricultur, fruit

Topic 7 Top Words:

Highest Prob: presid, american, peopl, will, one, countri, govern

FREX: immigr, polit, romney, voter, elect, campaign, obama

Lift: mitt, romney, poll, politician, gingrich, racism, racist

Score: obama, republican, romney, trump, democrat, polit, vote

Topic 8 Top Words:

Highest Prob: said, get, say, peopl, year, one, work

FREX: got, mother, son, daughter, didnt, apart, feel

Lift: shes, grandmoth, smile, neediest, couch, dad, daughter

Score: said, say, get, dont, got, just, daughter

> summary(bias_snap8)

A topic model with 8 topics, 11984 documents and a 4177 word dictionary.

Topic 1 Top Words:

Highest Prob: presid, said, republican, polit, democrat, american, campaign

FREX: trump, polit, dole, candid, elect, romney, voter

Lift: santorum, elector, mitt, romney, strategist, pollster, incumb

Score: republican, democrat, romney, obama, dole, clinton, senat

Topic 2 Top Words:

Highest Prob: said, citi, state, new, offici, servic, offic

FREX: mayor, citi, council, lawyer, court, file, offici

Lift: plaintiff, lawsuit, improp, fingerprint, bloomberg, rico, puerto

Score: citi, immigr, offici, mayor, court, agenc, depart

Topic 3 Top Words:

Highest Prob: welfar, program, state, children, work, benefit, peopl

FREX: welfar, recipi, benefit, child, elig, assist, program

Lift: welfare--work, able-bodi, afdc, caseload, --wedlock, unmarri, childless

Score: welfar, children, recipi, program, state, benefit, reform

Topic 4 Top Words:

Highest Prob: percent, year, incom, job, poverti, rate, increas

FREX: wage, rate, economi, percent, poverti, unemploy, minimum

Lift: economist, census, stagnat, inequ, wage, richest, capita

Score: percent, poverti, incom, wage, economi, econom, economist

Topic 5 Top Words:

Highest Prob: counti, block, polic, will, two, school, resid

FREX: stolen, polic, arrest, prison, sentenc, vehicl, male

Lift: ave, baton, robberi, stolen, honolulu, roug, lane

Score: stolen, block, counti, polic, ave, arrest, man

Topic 6 Top Words:

Highest Prob: said, famili, new, children, help, york, month

FREX: daughter, neediest, donat, brooklyn, chariti, rent, son

Lift: attn, payabl, schermerhorn, two-bedroom, one-bedroom, archdioces, joralemon

Score: neediest, brooklyn, mother, children, famili, daughter, chariti

Topic 7 Top Words:

Highest Prob: one, say, peopl, get, like, just, can

FREX: tell, know, didnt, stori, your, got, ive

Lift: yeah, gonna, okay, aint, cup, hey, funni

Score: know, think, kid, like, dont, man, thing

Topic 8 Top Words:

Highest Prob: bill, budget, tax, cut, billion, hous, program

FREX: billion, budget, bill, deficit, farm, medicar, cut

Lift: discretionari, across--board, r-kan, r-ga, r-ohio, billion, veto

Score: billion, republican, senat, budget, tax, democrat, vote

> summary(online_snap8)

A topic model with 8 topics, 3799 documents and a 2843 word dictionary.

Topic 1 Top Words:

Highest Prob: stamp, fraud, said, card, benefit, charg, store

FREX: fraud, investig, jeff, alleg, attorney, prison, arrest

Lift: conspir, lds, lyle, marijuana, mormon, polygamist, theft

Score: fraud, flds, prosecutor, indict, investig, lyle, card

Topic 2 Top Words:

Highest Prob: said, school, children, hunger, citi, meal, counti

FREX: student, hunger, school, meal, pantri, insecur, lunch

Lift: librari, student, campus, elementari, lunch, pantri, thanksgiv

Score: school, pantri, student, insecur, hunger, meal, counti

Topic 3 Top Words:

Highest Prob: market, snap, store, program, farmer, purchas, healthi

FREX: fruit, veget, market, obes, fresh, healthi, soda

Lift: dietari, fruit, junk, poultri, calori, desert, shopper

Score: farmer, store, veget, market, fruit, healthi, obes

Topic 4 Top Words:

Highest Prob: state, stamp, work, said, requir, peopl, benefit

FREX: main, requir, waiver, able-bodi, test, train, governor

Lift: abawd, reinstat, able-bodi, job-train, three-month, waiver, lepag

Score: waiver, able-bodi, welfar, job, work, requir, unemploy

Topic 5 Top Words:

Highest Prob: bill, cut, republican, stamp, program, hous, farm

FREX: democrat, republican, senat, vote, bill, trump, ryan

Lift: mcgovern, boehner, gop, senat, vote, cotton, gingrich

Score: republican, farm, bill, vote, democrat, senat, cut

Topic 6 Top Words:

Highest Prob: poverti, percent, american, incom, tax, year, rate

FREX: poverti, wage, tax, earn, rate, econom, minimum

Lift: eitc, index, gdp, means-test, median, census, poverti

Score: poverti, wage, welfar, rate, percent, incom, minimum

Topic 7 Top Words:

Highest Prob: program, benefit, state, assist, famili, snap, feder

FREX: medicaid, elig, immigr, care, servic, grant, militari

Lift: refuge, diaper, ssi, immigr, spous, militari, foster

Score: medicaid, elig, servic, benefit, refuge, famili, wic

Topic 8 Top Words:

Highest Prob: peopl, get, like, make, just, one, work

FREX: thing, think, stori, know, realli, dont, tell

Lift: mcdonald, mayb, youd, sick, ive, paltrow, ridicul

Score: job, walmart, paltrow, hour, work, dont, think
