# Supplementary material for: SNAP judgments into the digital age: Reporting on food stamps varies significantly with time, publication type, and political leaning
Source: PLoS One. 2020 Feb 21;15(2):e0229180. doi: 10.1371/journal.pone.0229180 (PMC7034891; doi:10.1371/journal.pone.0229180)
Supplement: S2 Appendix — (DOCX) [file pone.0229180.s002.docx]

> ###

> ## examine the outputs of estimateEffect models for print subset and online corpora, which have political alignment scores

> ## data used to generate Fig3

> ###

> summary(full_snap8_effect2)

Call:

estimateEffect(formula = 1:8 ~ s(yearmonth) + majorYN, stmobj = full_snap8,

metadata = PrintCorpusFull_out$meta)

Topic 1:

Coefficients:

Estimate Std. Error t value Pr(>|t|)

(Intercept) 0.054005 0.008406 6.424 1.33e-10 ***

s(yearmonth)1 -0.022459 0.014776 -1.520 0.12852

s(yearmonth)2 -0.005390 0.009216 -0.585 0.55863

s(yearmonth)3 -0.014155 0.011634 -1.217 0.22374

s(yearmonth)4 -0.019823 0.009134 -2.170 0.02999 *

s(yearmonth)5 -0.037631 0.009651 -3.899 9.67e-05 ***

s(yearmonth)6 -0.026989 0.009710 -2.779 0.00545 **

s(yearmonth)7 0.104180 0.009547 10.913 < 2e-16 ***

s(yearmonth)8 0.083775 0.011105 7.544 4.62e-14 ***

s(yearmonth)9 -0.060923 0.015409 -3.954 7.71e-05 ***

s(yearmonth)10 0.301840 0.037825 7.980 1.49e-15 ***

majorYNother_paper 0.043754 0.002008 21.792 < 2e-16 ***

---

Signif. codes: 0 ‘***’ 0.001 ‘**’ 0.01 ‘*’ 0.05 ‘.’ 0.1 ‘ ’ 1

Topic 2:

Coefficients:

Estimate Std. Error t value Pr(>|t|)

(Intercept) 0.214952 0.009718 22.120 < 2e-16 ***

s(yearmonth)1 -0.027722 0.016895 -1.641 0.101

s(yearmonth)2 0.044484 0.010681 4.165 3.12e-05 ***

s(yearmonth)3 -0.073462 0.013117 -5.601 2.15e-08 ***

s(yearmonth)4 0.047689 0.010583 4.506 6.61e-06 ***

s(yearmonth)5 -0.017037 0.010981 -1.551 0.121

s(yearmonth)6 -0.057558 0.011220 -5.130 2.91e-07 ***

s(yearmonth)7 -0.010224 0.011077 -0.923 0.356

s(yearmonth)8 -0.063596 0.012872 -4.941 7.80e-07 ***

s(yearmonth)9 -0.020019 0.017355 -1.153 0.249

s(yearmonth)10 -0.035577 0.039697 -0.896 0.370

majorYNother_paper -0.017655 0.002461 -7.174 7.38e-13 ***

---

Signif. codes: 0 ‘***’ 0.001 ‘**’ 0.01 ‘*’ 0.05 ‘.’ 0.1 ‘ ’ 1

Topic 3:

Coefficients:

Estimate Std. Error t value Pr(>|t|)

(Intercept) 0.073413 0.009781 7.506 6.19e-14 ***

s(yearmonth)1 -0.057853 0.017045 -3.394 0.000689 ***

s(yearmonth)2 -0.036949 0.010500 -3.519 0.000434 ***

s(yearmonth)3 -0.015833 0.013450 -1.177 0.239148

s(yearmonth)4 0.024853 0.010490 2.369 0.017835 *

s(yearmonth)5 0.006142 0.011106 0.553 0.580234

s(yearmonth)6 -0.024337 0.011164 -2.180 0.029272 *

s(yearmonth)7 -0.038925 0.010978 -3.546 0.000392 ***

s(yearmonth)8 0.026733 0.012754 2.096 0.036078 *

s(yearmonth)9 -0.010435 0.016857 -0.619 0.535883

s(yearmonth)10 -0.136022 0.037137 -3.663 0.000250 ***

majorYNother_paper 0.056985 0.002217 25.707 < 2e-16 ***

---

Signif. codes: 0 ‘***’ 0.001 ‘**’ 0.01 ‘*’ 0.05 ‘.’ 0.1 ‘ ’ 1

Topic 4:

Coefficients:

Estimate Std. Error t value Pr(>|t|)

(Intercept) 0.093819 0.009691 9.681 < 2e-16 ***

s(yearmonth)1 0.152022 0.017057 8.912 < 2e-16 ***

s(yearmonth)2 0.077443 0.010371 7.467 8.29e-14 ***

s(yearmonth)3 0.043872 0.013010 3.372 0.000746 ***

s(yearmonth)4 0.082876 0.010483 7.906 2.70e-15 ***

s(yearmonth)5 0.067968 0.010829 6.277 3.48e-10 ***

s(yearmonth)6 0.113968 0.011342 10.048 < 2e-16 ***

s(yearmonth)7 0.047661 0.010883 4.379 1.19e-05 ***

s(yearmonth)8 0.003521 0.012122 0.290 0.771443

s(yearmonth)9 0.155310 0.015681 9.905 < 2e-16 ***

s(yearmonth)10 0.026274 0.037258 0.705 0.480698

majorYNother_paper -0.049042 0.002134 -22.980 < 2e-16 ***

---

Signif. codes: 0 ‘***’ 0.001 ‘**’ 0.01 ‘*’ 0.05 ‘.’ 0.1 ‘ ’ 1

Topic 5:

Coefficients:

Estimate Std. Error t value Pr(>|t|)

(Intercept) 0.0849452 0.0081241 10.456 < 2e-16 ***

s(yearmonth)1 -0.0159029 0.0140590 -1.131 0.25799

s(yearmonth)2 0.0044439 0.0091847 0.484 0.62850

s(yearmonth)3 -0.0023173 0.0111651 -0.208 0.83558

s(yearmonth)4 -0.0441287 0.0089308 -4.941 7.79e-07 ***

s(yearmonth)5 -0.0169830 0.0093221 -1.822 0.06849 .

s(yearmonth)6 -0.0250048 0.0095291 -2.624 0.00869 **

s(yearmonth)7 -0.0225587 0.0093845 -2.404 0.01623 *

s(yearmonth)8 0.0082961 0.0104772 0.792 0.42847

s(yearmonth)9 0.0005206 0.0148303 0.035 0.97199

s(yearmonth)10 0.0242423 0.0328446 0.738 0.46046

majorYNother_paper 0.0098681 0.0018837 5.239 1.62e-07 ***

---

Signif. codes: 0 ‘***’ 0.001 ‘**’ 0.01 ‘*’ 0.05 ‘.’ 0.1 ‘ ’ 1

Topic 6:

Coefficients:

Estimate Std. Error t value Pr(>|t|)

(Intercept) 0.051335 0.006036 8.505 < 2e-16 ***

s(yearmonth)1 -0.019573 0.010537 -1.857 0.063245 .

s(yearmonth)2 -0.016739 0.006513 -2.570 0.010168 *

s(yearmonth)3 -0.020293 0.008346 -2.431 0.015041 *

s(yearmonth)4 0.009413 0.006512 1.445 0.148331

s(yearmonth)5 -0.007992 0.007207 -1.109 0.267437

s(yearmonth)6 0.013028 0.006965 1.870 0.061422 .

s(yearmonth)7 0.033186 0.006904 4.806 1.54e-06 ***

s(yearmonth)8 0.009075 0.008133 1.116 0.264480

s(yearmonth)9 0.036877 0.011083 3.327 0.000878 ***

s(yearmonth)10 0.010054 0.027091 0.371 0.710551

majorYNother_paper 0.015172 0.001473 10.301 < 2e-16 ***

---

Signif. codes: 0 ‘***’ 0.001 ‘**’ 0.01 ‘*’ 0.05 ‘.’ 0.1 ‘ ’ 1

Topic 7:

Coefficients:

Estimate Std. Error t value Pr(>|t|)

(Intercept) 0.131192 0.010047 13.058 < 2e-16 ***

s(yearmonth)1 0.097272 0.018224 5.337 9.46e-08 ***

s(yearmonth)2 0.019264 0.010650 1.809 0.0705 .

s(yearmonth)3 0.062829 0.013706 4.584 4.57e-06 ***

s(yearmonth)4 -0.015711 0.010547 -1.490 0.1363

s(yearmonth)5 0.066156 0.011558 5.724 1.05e-08 ***

s(yearmonth)6 0.156458 0.011070 14.134 < 2e-16 ***

s(yearmonth)7 0.005119 0.011603 0.441 0.6591

s(yearmonth)8 0.080967 0.012844 6.304 2.92e-10 ***

s(yearmonth)9 0.009265 0.016713 0.554 0.5793

s(yearmonth)10 0.078376 0.038727 2.024 0.0430 *

majorYNother_paper -0.034384 0.002329 -14.762 < 2e-16 ***

---

Signif. codes: 0 ‘***’ 0.001 ‘**’ 0.01 ‘*’ 0.05 ‘.’ 0.1 ‘ ’ 1

Topic 8:

Coefficients:

Estimate Std. Error t value Pr(>|t|)

(Intercept) 0.296481 0.010503 28.228 < 2e-16 ***

s(yearmonth)1 -0.105838 0.018426 -5.744 9.30e-09 ***

s(yearmonth)2 -0.086983 0.011484 -7.574 3.67e-14 ***

s(yearmonth)3 0.019397 0.014266 1.360 0.174

s(yearmonth)4 -0.085525 0.011362 -7.527 5.27e-14 ***

s(yearmonth)5 -0.060706 0.012061 -5.033 4.84e-07 ***

s(yearmonth)6 -0.149839 0.012101 -12.383 < 2e-16 ***

s(yearmonth)7 -0.118429 0.012282 -9.642 < 2e-16 ***

s(yearmonth)8 -0.149153 0.013281 -11.231 < 2e-16 ***

s(yearmonth)9 -0.110782 0.017982 -6.161 7.29e-10 ***

s(yearmonth)10 -0.270131 0.039871 -6.775 1.25e-11 ***

majorYNother_paper -0.024624 0.002487 -9.899 < 2e-16 ***

---

Signif. codes: 0 ‘***’ 0.001 ‘**’ 0.01 ‘*’ 0.05 ‘.’ 0.1 ‘ ’ 1

> summary(bias_snap8_effect3)

Call:

estimateEffect(formula = 1:8 ~ s(yearmonth) + avg_align, stmobj = bias_snap8,

metadata = PrintCorpusBias_out$meta)

Topic 1:

Coefficients:

Estimate Std. Error t value Pr(>|t|)

(Intercept) 0.072091 0.015064 4.786 1.72e-06 ***

s(yearmonth)1 0.060681 0.030009 2.022 0.043190 *

s(yearmonth)2 0.014117 0.018070 0.781 0.434691

s(yearmonth)3 0.082514 0.018552 4.448 8.76e-06 ***

s(yearmonth)4 0.002233 0.017708 0.126 0.899650

s(yearmonth)5 0.084111 0.020092 4.186 2.86e-05 ***

s(yearmonth)6 -0.066203 0.018465 -3.585 0.000338 ***

s(yearmonth)7 0.145386 0.017942 8.103 5.87e-16 ***

s(yearmonth)8 0.019190 0.021168 0.907 0.364665

s(yearmonth)9 0.086507 0.026352 3.283 0.001031 **

s(yearmonth)10 0.100302 0.048198 2.081 0.037452 *

avg_align 0.014928 0.008460 1.765 0.077655 .

---

Signif. codes: 0 ‘***’ 0.001 ‘**’ 0.01 ‘*’ 0.05 ‘.’ 0.1 ‘ ’ 1

Topic 2:

Coefficients:

Estimate Std. Error t value Pr(>|t|)

(Intercept) 0.151358 0.015437 9.805 < 2e-16 ***

s(yearmonth)1 0.035924 0.029665 1.211 0.225937

s(yearmonth)2 -0.011686 0.017552 -0.666 0.505566

s(yearmonth)3 -0.044522 0.018405 -2.419 0.015577 *

s(yearmonth)4 0.060580 0.017914 3.382 0.000723 ***

s(yearmonth)5 -0.049803 0.020128 -2.474 0.013363 *

s(yearmonth)6 0.026417 0.019040 1.387 0.165335

s(yearmonth)7 -0.061708 0.017379 -3.551 0.000386 ***

s(yearmonth)8 0.016656 0.021484 0.775 0.438186

s(yearmonth)9 -0.044824 0.025438 -1.762 0.078083 .

s(yearmonth)10 -0.012872 0.047466 -0.271 0.786259

avg_align 0.028364 0.008026 3.534 0.000411 ***

---

Signif. codes: 0 ‘***’ 0.001 ‘**’ 0.01 ‘*’ 0.05 ‘.’ 0.1 ‘ ’ 1

Topic 3:

Coefficients:

Estimate Std. Error t value Pr(>|t|)

(Intercept) 0.1285935 0.0152921 8.409 < 2e-16 ***

s(yearmonth)1 0.0409496 0.0294656 1.390 0.1646

s(yearmonth)2 0.0005664 0.0178740 0.032 0.9747

s(yearmonth)3 0.1399612 0.0194803 7.185 7.13e-13 ***

s(yearmonth)4 -0.0050526 0.0172878 -0.292 0.7701

s(yearmonth)5 -0.0081836 0.0206963 -0.395 0.6925

s(yearmonth)6 -0.0130139 0.0185972 -0.700 0.4841

s(yearmonth)7 -0.0252640 0.0184248 -1.371 0.1703

s(yearmonth)8 -0.0134346 0.0210584 -0.638 0.5235

s(yearmonth)9 -0.0720548 0.0283645 -2.540 0.0111 *

s(yearmonth)10 0.1293787 0.0527795 2.451 0.0142 *

avg_align 0.0073420 0.0088063 0.834 0.4045

---

Signif. codes: 0 ‘***’ 0.001 ‘**’ 0.01 ‘*’ 0.05 ‘.’ 0.1 ‘ ’ 1

Topic 4:

Coefficients:

Estimate Std. Error t value Pr(>|t|)

(Intercept) 0.159752 0.016152 9.890 < 2e-16 ***

s(yearmonth)1 -0.053125 0.030001 -1.771 0.0766 .

s(yearmonth)2 -0.005216 0.017382 -0.300 0.7641

s(yearmonth)3 -0.104726 0.019564 -5.353 8.81e-08 ***

s(yearmonth)4 -0.016162 0.017729 -0.912 0.3620

s(yearmonth)5 -0.127802 0.021470 -5.952 2.72e-09 ***

s(yearmonth)6 0.043500 0.019763 2.201 0.0277 *

s(yearmonth)7 -0.027812 0.018978 -1.466 0.1428

s(yearmonth)8 0.032493 0.021432 1.516 0.1295

s(yearmonth)9 -0.036392 0.028434 -1.280 0.2006

s(yearmonth)10 -0.038315 0.055469 -0.691 0.4897

avg_align -0.009498 0.008634 -1.100 0.2713

---

Signif. codes: 0 ‘***’ 0.001 ‘**’ 0.01 ‘*’ 0.05 ‘.’ 0.1 ‘ ’ 1

Topic 5:

Coefficients:

Estimate Std. Error t value Pr(>|t|)

(Intercept) 0.108698 0.011796 9.215 <2e-16 ***

s(yearmonth)1 0.030855 0.022803 1.353 0.1760

s(yearmonth)2 0.001434 0.013569 0.106 0.9158

s(yearmonth)3 -0.036072 0.014551 -2.479 0.0132 *

s(yearmonth)4 0.004989 0.013823 0.361 0.7182

s(yearmonth)5 0.007171 0.016685 0.430 0.6673

s(yearmonth)6 -0.027496 0.014418 -1.907 0.0565 .

s(yearmonth)7 -0.022662 0.014081 -1.609 0.1075

s(yearmonth)8 -0.002807 0.016601 -0.169 0.8657

s(yearmonth)9 0.009997 0.021719 0.460 0.6453

s(yearmonth)10 -0.096074 0.039853 -2.411 0.0159 *

avg_align 0.076904 0.006496 11.838 <2e-16 ***

---

Signif. codes: 0 ‘***’ 0.001 ‘**’ 0.01 ‘*’ 0.05 ‘.’ 0.1 ‘ ’ 1

Topic 6:

Coefficients:

Estimate Std. Error t value Pr(>|t|)

(Intercept) 0.078103 0.017709 4.410 1.04e-05 ***

s(yearmonth)1 -0.054652 0.034489 -1.585 0.1131

s(yearmonth)2 -0.008718 0.020298 -0.429 0.6676

s(yearmonth)3 -0.093988 0.021946 -4.283 1.86e-05 ***

s(yearmonth)4 0.032321 0.020538 1.574 0.1156

s(yearmonth)5 0.048026 0.023953 2.005 0.0450 *

s(yearmonth)6 0.029697 0.022177 1.339 0.1806

s(yearmonth)7 -0.037933 0.020792 -1.824 0.0681 .

s(yearmonth)8 -0.039831 0.024533 -1.624 0.1045

s(yearmonth)9 0.063317 0.031764 1.993 0.0462 *

s(yearmonth)10 -0.142272 0.058937 -2.414 0.0158 *

avg_align -0.174771 0.009835 -17.770 < 2e-16 ***

---

Signif. codes: 0 ‘***’ 0.001 ‘**’ 0.01 ‘*’ 0.05 ‘.’ 0.1 ‘ ’ 1

Topic 7:

Coefficients:

Estimate Std. Error t value Pr(>|t|)

(Intercept) 0.1527443 0.0152169 10.038 < 2e-16 ***

s(yearmonth)1 -0.0386399 0.0297763 -1.298 0.19442

s(yearmonth)2 0.0315530 0.0176451 1.788 0.07377 .

s(yearmonth)3 -0.0333396 0.0183719 -1.815 0.06959 .

s(yearmonth)4 0.0211411 0.0174756 1.210 0.22640

s(yearmonth)5 0.0003777 0.0215173 0.018 0.98599

s(yearmonth)6 0.0515817 0.0186397 2.767 0.00566 **

s(yearmonth)7 0.0097955 0.0179875 0.545 0.58606

s(yearmonth)8 0.0483226 0.0217085 2.226 0.02603 *

s(yearmonth)9 0.0780175 0.0280994 2.776 0.00550 **

s(yearmonth)10 -0.0462547 0.0514192 -0.900 0.36837

avg_align 0.0201409 0.0087573 2.300 0.02147 *

---

Signif. codes: 0 ‘***’ 0.001 ‘**’ 0.01 ‘*’ 0.05 ‘.’ 0.1 ‘ ’ 1

Topic 8:

Coefficients:

Estimate Std. Error t value Pr(>|t|)

(Intercept) 0.14857 0.01767 8.406 < 2e-16 ***

s(yearmonth)1 -0.02192 0.03362 -0.652 0.514451

s(yearmonth)2 -0.02185 0.02050 -1.065 0.286671

s(yearmonth)3 0.09039 0.02152 4.199 2.69e-05 ***

s(yearmonth)4 -0.09952 0.02054 -4.845 1.28e-06 ***

s(yearmonth)5 0.04605 0.02406 1.914 0.055694 .

s(yearmonth)6 -0.04443 0.02243 -1.981 0.047630 *

s(yearmonth)7 0.02047 0.02095 0.977 0.328596

s(yearmonth)8 -0.06052 0.02440 -2.480 0.013143 *

s(yearmonth)9 -0.08391 0.03299 -2.543 0.010988 *

s(yearmonth)10 0.10538 0.05996 1.757 0.078859 .

avg_align 0.03690 0.01009 3.658 0.000256 ***

---

Signif. codes: 0 ‘***’ 0.001 ‘**’ 0.01 ‘*’ 0.05 ‘.’ 0.1 ‘ ’ 1

> summary(online_snap8_effect5)

Call:

estimateEffect(formula = 1:8 ~ s(yearmonth) + majorYN + avg_align,

stmobj = online_snap8, metadata = OnlineCorpus_out$meta)

Topic 1:

Coefficients:

Estimate Std. Error t value Pr(>|t|)

(Intercept) 0.030206 0.102122 0.296 0.76741

s(yearmonth)1 0.016733 0.184743 0.091 0.92784

s(yearmonth)2 0.110690 0.102582 1.079 0.28064

s(yearmonth)3 0.058155 0.107603 0.540 0.58891

s(yearmonth)4 0.148496 0.103762 1.431 0.15248

s(yearmonth)5 0.048925 0.106820 0.458 0.64697

s(yearmonth)6 0.139444 0.105833 1.318 0.18772

s(yearmonth)7 0.075426 0.103973 0.725 0.46823

s(yearmonth)8 0.358597 0.115853 3.095 0.00198 **

s(yearmonth)9 -0.292502 0.165911 -1.763 0.07798 .

s(yearmonth)10 0.496216 0.200491 2.475 0.01337 *

majorYN1 0.020863 0.010311 2.023 0.04311 *

avg_align 0.064059 0.008059 7.948 2.47e-15 ***

---

Signif. codes: 0 ‘***’ 0.001 ‘**’ 0.01 ‘*’ 0.05 ‘.’ 0.1 ‘ ’ 1

Topic 2:

Coefficients:

Estimate Std. Error t value Pr(>|t|)

(Intercept) 0.220765 0.083571 2.642 0.00828 **

s(yearmonth)1 -0.158781 0.143826 -1.104 0.26967

s(yearmonth)2 -0.122484 0.081067 -1.511 0.13090

s(yearmonth)3 -0.141795 0.088184 -1.608 0.10793

s(yearmonth)4 -0.098173 0.084542 -1.161 0.24562

s(yearmonth)5 -0.160894 0.086374 -1.863 0.06257 .

s(yearmonth)6 -0.129644 0.085202 -1.522 0.12819

s(yearmonth)7 -0.122306 0.085781 -1.426 0.15401

s(yearmonth)8 -0.133195 0.090678 -1.469 0.14195

s(yearmonth)9 -0.080965 0.124465 -0.650 0.51541

s(yearmonth)10 -0.069197 0.141833 -0.488 0.62567

majorYN1 0.047752 0.007229 6.605 4.52e-11 ***

avg_align -0.050302 0.005371 -9.366 < 2e-16 ***

---

Signif. codes: 0 ‘***’ 0.001 ‘**’ 0.01 ‘*’ 0.05 ‘.’ 0.1 ‘ ’ 1

Topic 3:

Coefficients:

Estimate Std. Error t value Pr(>|t|)

(Intercept) 0.012691 0.076627 0.166 0.8685

s(yearmonth)1 0.208440 0.142353 1.464 0.1432

s(yearmonth)2 0.077479 0.078025 0.993 0.3208

s(yearmonth)3 0.076971 0.080686 0.954 0.3402

s(yearmonth)4 0.058609 0.077907 0.752 0.4519

s(yearmonth)5 0.135949 0.079162 1.717 0.0860 .

s(yearmonth)6 0.034487 0.080626 0.428 0.6689

s(yearmonth)7 0.102737 0.077539 1.325 0.1853

s(yearmonth)8 0.072612 0.088565 0.820 0.4123

s(yearmonth)9 0.295012 0.126562 2.331 0.0198 *

s(yearmonth)10 0.021325 0.144154 0.148 0.8824

majorYN1 -0.009453 0.007946 -1.190 0.2342

avg_align -0.013524 0.006082 -2.224 0.0262 *

---

Signif. codes: 0 ‘***’ 0.001 ‘**’ 0.01 ‘*’ 0.05 ‘.’ 0.1 ‘ ’ 1

Topic 4:

Coefficients:

Estimate Std. Error t value Pr(>|t|)

(Intercept) 8.823e-02 7.919e-02 1.114 0.2653

s(yearmonth)1 3.947e-03 1.449e-01 0.027 0.9783

s(yearmonth)2 1.092e-02 7.943e-02 0.137 0.8907

s(yearmonth)3 3.719e-03 8.283e-02 0.045 0.9642

s(yearmonth)4 9.202e-02 8.085e-02 1.138 0.2551

s(yearmonth)5 -3.162e-05 8.296e-02 0.000 0.9997

s(yearmonth)6 1.742e-01 8.162e-02 2.134 0.0329 *

s(yearmonth)7 9.624e-02 8.095e-02 1.189 0.2346

s(yearmonth)8 -4.946e-02 8.955e-02 -0.552 0.5808

s(yearmonth)9 1.432e-01 1.315e-01 1.089 0.2762

s(yearmonth)10 -2.371e-02 1.422e-01 -0.167 0.8676

majorYN1 2.236e-02 7.997e-03 2.796 0.0052 **

avg_align 5.767e-02 6.366e-03 9.059 <2e-16 ***

---

Signif. codes: 0 ‘***’ 0.001 ‘**’ 0.01 ‘*’ 0.05 ‘.’ 0.1 ‘ ’ 1

Topic 5:

Coefficients:

Estimate Std. Error t value Pr(>|t|)

(Intercept) 0.129173 0.072620 1.779 0.0754 .

s(yearmonth)1 -0.107825 0.134140 -0.804 0.4215

s(yearmonth)2 0.050888 0.075309 0.676 0.4993

s(yearmonth)3 0.069033 0.076398 0.904 0.3663

s(yearmonth)4 -0.081168 0.073186 -1.109 0.2675

s(yearmonth)5 -0.007507 0.076097 -0.099 0.9214

s(yearmonth)6 -0.068832 0.074569 -0.923 0.3560

s(yearmonth)7 -0.026527 0.074201 -0.357 0.7207

s(yearmonth)8 -0.130564 0.081615 -1.600 0.1097

s(yearmonth)9 0.236420 0.114365 2.067 0.0388 *

s(yearmonth)10 -0.207843 0.129307 -1.607 0.1081

majorYN1 -0.014053 0.007307 -1.923 0.0545 .

avg_align -0.031563 0.005395 -5.850 5.32e-09 ***

---

Signif. codes: 0 ‘***’ 0.001 ‘**’ 0.01 ‘*’ 0.05 ‘.’ 0.1 ‘ ’ 1

Topic 6:

Coefficients:

Estimate Std. Error t value Pr(>|t|)

(Intercept) 0.229849 0.077892 2.951 0.003188 **

s(yearmonth)1 -0.044459 0.139077 -0.320 0.749233

s(yearmonth)2 -0.138461 0.076171 -1.818 0.069179 .

s(yearmonth)3 -0.070257 0.082020 -0.857 0.391733

s(yearmonth)4 -0.083667 0.078692 -1.063 0.287750

s(yearmonth)5 -0.089976 0.080934 -1.112 0.266325

s(yearmonth)6 -0.114888 0.079917 -1.438 0.150633

s(yearmonth)7 -0.098986 0.078442 -1.262 0.207066

s(yearmonth)8 -0.099513 0.087392 -1.139 0.254904

s(yearmonth)9 -0.175136 0.118037 -1.484 0.137964

s(yearmonth)10 -0.206850 0.132482 -1.561 0.118527

majorYN1 -0.028518 0.007541 -3.782 0.000158 ***

avg_align -0.011736 0.005703 -2.058 0.039683 *

---

Signif. codes: 0 ‘***’ 0.001 ‘**’ 0.01 ‘*’ 0.05 ‘.’ 0.1 ‘ ’ 1

Topic 7:

Coefficients:

Estimate Std. Error t value Pr(>|t|)

(Intercept) 0.093062 0.060032 1.550 0.1212

s(yearmonth)1 0.043859 0.108888 0.403 0.6871

s(yearmonth)2 0.058929 0.059572 0.989 0.3226

s(yearmonth)3 0.016216 0.064136 0.253 0.8004

s(yearmonth)4 0.049826 0.061516 0.810 0.4180

s(yearmonth)5 0.016112 0.061345 0.263 0.7928

s(yearmonth)6 0.050745 0.061663 0.823 0.4106

s(yearmonth)7 0.009860 0.061274 0.161 0.8722

s(yearmonth)8 0.074153 0.070378 1.054 0.2921

s(yearmonth)9 -0.093401 0.096700 -0.966 0.3342

s(yearmonth)10 0.190656 0.111835 1.705 0.0883 .

majorYN1 0.010116 0.005886 1.719 0.0858 .

avg_align 0.030702 0.004555 6.741 1.82e-11 ***

---

Signif. codes: 0 ‘***’ 0.001 ‘**’ 0.01 ‘*’ 0.05 ‘.’ 0.1 ‘ ’ 1

Topic 8:

Coefficients:

Estimate Std. Error t value Pr(>|t|)

(Intercept) 0.198173 0.073430 2.699 0.00699 **

s(yearmonth)1 0.036355 0.131370 0.277 0.78199

s(yearmonth)2 -0.051153 0.070878 -0.722 0.47052

s(yearmonth)3 -0.013754 0.076723 -0.179 0.85774

s(yearmonth)4 -0.088701 0.074856 -1.185 0.23611

s(yearmonth)5 0.054777 0.075729 0.723 0.46952

s(yearmonth)6 -0.086779 0.074924 -1.158 0.24684

s(yearmonth)7 -0.038908 0.074652 -0.521 0.60227

s(yearmonth)8 -0.093894 0.080501 -1.166 0.24354

s(yearmonth)9 -0.037750 0.112199 -0.336 0.73655

s(yearmonth)10 -0.199022 0.123651 -1.610 0.10758

majorYN1 -0.049095 0.006789 -7.232 5.75e-13 ***

avg_align -0.045185 0.005504 -8.210 3.01e-16 ***

---

Signif. codes: 0 ‘***’ 0.001 ‘**’ 0.01 ‘*’ 0.05 ‘.’ 0.1 ‘ ’ 1
